# Intein Inhibitors as Novel Antimicrobials: Protein Splicing in Human Pathogens, Screening Methods, and Off-Target Considerations

**DOI:** 10.3389/fmolb.2021.752824

**Published:** 2021-10-07

**Authors:** Diana A. Wall, Seanan P. Tarrant, Chunyu Wang, Kenneth V. Mills, Christopher W. Lennon

**Affiliations:** ^1^ Department of Chemistry, College of the Holy Cross, Worcester, MA, United States; ^2^ Department of Biological Sciences, Center for Biotechnology and Interdisciplinary Studies, Rensselaer Polytechnic Institute, Troy, NY, United States; ^3^ Department of Chemistry and Chemical Biology, Center for Biotechnology and Interdisciplinary Studies, Rensselaer Polytechnic Institute, Troy, NY, United States; ^4^ Department of Biological Sciences, Murray State University, Murray, KY, United States

**Keywords:** intein, protein splicing, novel drug target, inhibitor screening, tuberculosis, hedgehog signaling, hedgehog autoprocessing

## Abstract

Protein splicing is a post-translational process by which an intervening polypeptide, or intein, catalyzes its own removal from the flanking polypeptides, or exteins, concomitant with extein ligation. Although inteins are highly abundant in the microbial world, including within several human pathogens, they are absent in the genomes of metazoans. As protein splicing is required to permit function of essential proteins within pathogens, inteins represent attractive antimicrobial targets. Here we review key proteins interrupted by inteins in pathogenic mycobacteria and fungi, exciting discoveries that provide proof of concept that intein activity can be inhibited and that this inhibition has an effect on the host organism’s fitness, and bioanalytical methods that have been used to screen for intein activity. We also consider potential off-target inhibition of hedgehog signaling, given the similarity in structure and function of inteins and hedgehog autoprocessing domains.

## Introduction

Inteins are intervening polypeptides translated within host proteins that are removed through protein splicing. Although inteins are widespread in the microbial world (Novikova et al., 2016), they are not present in the genomes of metazoans. Given that several devastating human pathogens house inteins within proteins essential for survival, and intein removal is considered necessary to permit host protein function, inteins represent attractive novel drug targets (Gangopadhyay et al., 2003; Paulus, 2003; Grainger and Beggs, 2005).

**FIGURE 1:**
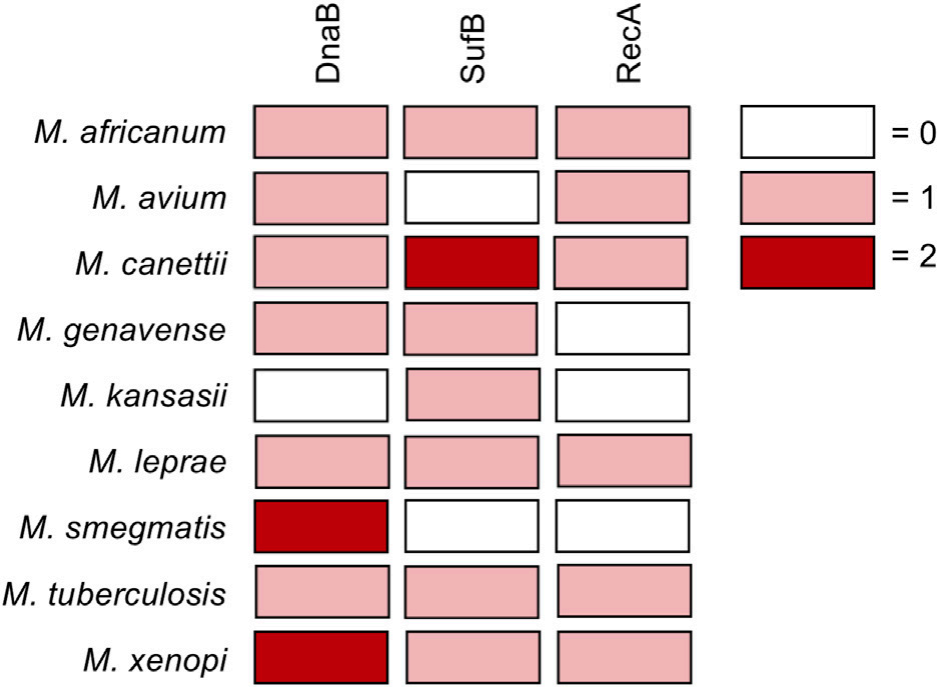
Inteins in DnaB, RecA, and SufB from pathogenic mycobacteria. Diagram showing inteins within the DnaB, RecA, and SufB genes of pathogenic mycobacteria. Boxes colored according to number of inteins in gene as follows: no intein in the gene, white; one intein in the gene, pink; two inteins in gene, maroon.

Of particular interest are mycobacterial pathogens including *Mycobacterium tuberculosis*, *Mycobacterium leprae*, and non-tuberculosis mycobacteria (NTM), which house inteins within important genes such as DnaB, RecA, SufB, and others (Figure 1). Prior to the recent Covid-19 pandemic, tuberculosis was the leading cause of death from an infectious agent worldwide, claiming 1.4 million deaths in 2019 (2020b). Additionally, a number of fungal pathogens such as *Cryptococcus neoformans*, *Cryptococcus gattii*, *Aspergillus fumigatus*, *Aspergillus nidulans*, and *Histoplasma capsulatum* also harbor inteins, most often in the essential spliceosomal protein Prp8 (Green et al., 2019). *C. neoformans* infections pose a particular threat to immunocompromised individuals such as those with HIV/AIDS, with an estimated 220,000 cases of cryptococcal meningitis occurring worldwide each year (Centers for Disease Control and Prevention, 2020).

Inteins are found in all three domains of life, as well in viruses, with the highest abundance within archaea and bacteria, with about 47 and 24% of species harboring inteins, respectively (Novikova et al., 2016). Only about 1% of the eukarya have inteins (Novikova et al., 2016). There is considerable evidence, although in some cases indirect, that supports the idea that blocking protein splicing will compromise host protein function. For example, inteins often localize to critical regions, such as the active site, of essential proteins involved in DNA replication, recombination, and repair (Novikova et al., 2014; Kelley et al., 2016; Novikova et al., 2016). If the intein is within an essential gene, as many are, inhibition of splicing should result in killing or prevention of reproduction of the host organism. However, as inteins have historically been studied in splicing reporters to study activity and not within the native extein context, there are not many examples of studies that monitor intein influence on host protein function; this is important future work needed in the field. However, some direct evidence of intein inhibition influencing host protein function does exist. One example of the link between splicing and host protein function is found in the intein-interrupted SufB protein of *M. tuberculosis*. SufB is part of the essential SUF [Fe-S] cluster biogenesis complex and interacts with SufC and SufD to form the SufBCD. Prior to splicing, the SufB intein was shown to block interactions with SufC and SufD (Huet et al., 2006). In the case of intein-interrupted RadA from the archaeon *Pyrococcus horikoshii*, which harbors an intein within the ATP-coordinating P-loop, ATPase activity is compromised prior to protein splicing (Topilina et al., 2015b). Recently, exciting work has demonstrated that selective inhibition of inteins interrupting the Prp8 protein in intein-containing fungal pathogens can influence survival of the organism (discussed below; (Li et al., 2021).

Here, we will discuss inteins as potential targets to selectively kill bacterial and fungal pathogens, along with the screening assays that could be used to test the ability of small molecules to inhibit protein splicing. There is proof of concept evidence that divalent cations (in particular zinc and copper), reactive oxygen, nitrogen, and chlorine derivatives, as well as small molecules including cisplatin and diethanolamine can act as splicing inhibitors and impact the fitness of pathogenic mycobacteria and fungi (Gangopadhyay et al., 2003; Zhang et al., 2010; Zhang et al., 2011; Dasari et al., 2015; Topilina et al., 2015a; Kelley et al., 2018; Li et al., 2019; Lennon et al., 2021; Panda et al., 2021) The discovery of drug-like molecules that can successfully inhibit the splicing of proteins essential for survival of pathogens could represent new treatment options. Because inteins are absent in humans, specific intein inhibitors could have limited side effects. The recent discovery of a small molecule inhibitor of the Prp8 intein that selectively inhibits splicing without targeting proteases and inhibits the growth of intein-containing *C. gattii* and *C. neoformans* is a particularly exciting breakthrough that suggests intein inhibitors may have therapeutic benefits (Li et al., 2021).

Reporter systems have been developed to detect intein activity and could be used as screening assays for potential drug leads. Here, we will discuss screening systems involving kanamycin resistance, green fluorescent protein (GFP), luciferase, CcdB/CcdA, thymidylate synthetase (TS), and β-galactosidase, and evaluate how they could be adapted to a drug screening system (Daugelat and Jacobs, 1999; Wood et al., 1999; Adam and Perler, 2002; Lew and Paulus, 2002; Gangopadhyay et al., 2003; Paulus, 2003; Buskirk et al., 2004; Zeidler et al., 2004; Bradley et al., 2005; Wong et al., 2005; Hiraga et al., 2009; Liang et al., 2011; Zhang et al., 2011; Chan et al., 2016; Neugebauer et al., 2017; Stevens et al., 2017; Kelley et al., 2018; Woods et al., 2020). Additionally, inteins and the metazoan Hedgehog (Hh) family of proteins are both HINT (Hedgehog-INTein) domain proteins with a similar fold (Porter et al., 1996a; Porter et al., 1996b; Hall et al., 1997). We therefore consider Hh autoprocessing, as the cholesterification process is similar mechanistically to protein splicing. Although the Hh pathway is relatively inactive in adults outside of stem cell maintenance, inhibition of the pathway could be a problematic unintended target of a potential intein-directed drug (Rimkus et al., 2016). On the other hand, autoprocessing inhibitors could be useful as a cancer therapy in certain basal cell carcinomas or medulloblastomas (Gupta et al., 2010).

## Mechanism of Protien Splicing, Intein Variety, and Possible Role as Environmental Sensors

Small molecules that target intein action have so far been shown to modify the active site nucleophiles of the intein active site. In this section we briefly review the mechanism of protein splicing, and in the sections that follow we discuss in detail how these steps are affected by known intein inhibitors.

Key conserved residues in most inteins include an N-terminal Cys or Ser and a C-terminal Asn. Inteins also require a Cys, Ser, or Thr as the first residue following the intein. Via protein splicing, inteins can self-catalyze their own excision from the two flanking polypeptide sequences, or exteins, to form a mature, functional host protein. The intein residues are numbered such that the N-terminal residue is Cys1 or Ser1 and continuing downstream, with the first C-extein residue as +1 and the last N-extein residue as -1.

**FIGURE 2:**
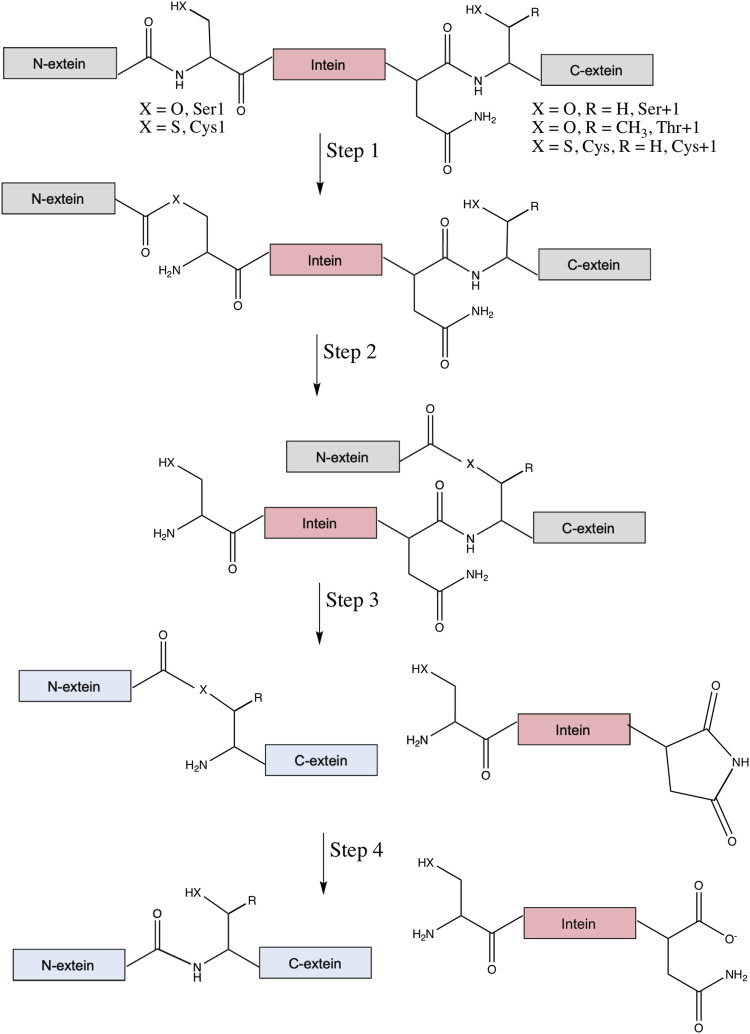
Canonical mechanism of protein splicing. Protein splicing is a post-translational process where an intein (in pink) interrupts the N- and C-exteins (in gray), which usually inactivates the extein’s native function. Step 1 is an amide-to-ester (or thioester) rearrangement of the peptide bond linking the N-extein and intein, facilitated by the N-terminal Ser1 or Cys1 of the intein (X = O for Ser, S for Cys). Step 2 is a trans-thioesterification reaction that transfers the N-extein from the side chain of the first intein residue to the side chain of the first C-extein residue, either Ser+1, Thr+1, or Cys+1 (X = O, R = H for Ser, X = S and R = H for Cys, and X = O and R = CH_3_ for Thr). Step 3 is cyclization of the C-terminal Asn (or sometimes Gln) of the intein coupled to peptide bond cleavage between the intein and C-extein. Linkage of the exteins and excision of the intein likely restores function to the exteins (now colored blue). Finally, step four converts the ester bond linking the exteins to the amide peptide bond and, in some cases, converts the C-terminal aminosuccinimide of the excised intein to Asn (or *iso*-Asn).

The canonical mechanism of protein splicing is a four-step process (Figure 2). First, the amide bond that connects the N-extein to the intein is rearranged to an ester or thioester, with the N-terminal Ser or Cys of the intein as the nucleophile. Then, transesterification via nucleophilic attack by the first residue of the C-extein (Cys, Ser, or Thr) on the ester formed in step one results in a branched intermediate, with the N-extein moved from the side chain of the first intein residue to the side chain of the first C-extein residue. These reactive nucleophiles are the target of most intein inhibitors discussed below. It will be essential that future lead compounds are selected that bind selectively to inteins. Given the long history of the development of selective protease inhibitors in therapy (Sojka et al., 2021), and that inteins promote similar chemical steps despite serving as their own substrate (Paulus, 2001), it seems possible that traditional pharmacological strategies would be useful for increasing selectivity of intein inhibitors as well.

Next, in the third step of splicing the branched ester or thioester is resolved by asparagine cyclization coupled to peptide bond cleavage between the intein and C-extein. The final step converts the ester bond linking the exteins to an amide bond, as well as hydrolysis of the C-terminal aminosuccinimide of the intein to Asn or *iso*-Asn (Mills et al., 2014; Shah and Muir, 2014). Two alternative classes of splicing also exist, with key differences reviewed elsewhere (Mills et al., 2014). Irreversible off-pathway reactions can also occur, where either the N- or C-extein is cleaved from the intein prior to ligation. With the ability of inteins to rearrange and cleave peptide bonds, they have been utilized extensively in biotechnology, a topic covered in several other comprehensive reviews (Volkmann and Mootz, 2013; Shah and Muir, 2014; Topilina and Mills, 2014; Wood and Camarero, 2014; Debelouchina and Muir, 2017; Sarmiento and Camarero, 2019; Nanda et al., 2020; Romero-Casanas et al., 2020).

Inteins come in a few different varieties. Full-length inteins contain a nested homing endonuclease (HE) domain within the autoprocessing domain. The HE domain can facilitate intein homing into intein-less alleles by making a double strand break in the extein sequence that lacks the interrupting intein gene, followed by DNA repair via homologous recombination using the intein-containing allele as a template. Mini-inteins, which lack the HE domain but retain the autoprocessing domain, are also abundant. In both cases, these contiguous inteins facilitate splicing in *cis* (Mills et al., 2014)*.* In contrast, some inteins are naturally split, with the autoprocessing domain expressed as two separate polypeptides that must first assemble before protein splicing occurs in *trans* (Volkmann and Iwai, 2010; Aranko et al., 2014; Li, 2015).

Although inteins don’t require any cofactor or chaperone to splice (Mills et al., 2014), the rate and accuracy of the reaction can be highly dependent on environmental conditions, including redox state, pH, salt concentration, and temperature (Belfort, 2017; Lennon and Belfort, 2017). As many inteins also are mobile genetic elements, they have traditionally been viewed as selfish molecular parasites (Gogarten and Hilario, 2006). However, several recent studies have provided evidence that some inteins may play a beneficial role for the host organism by providing post-translational control of key enzymes in response to stress (Belfort, 2017; Lennon and Belfort, 2017).

## Intein Presence in Human Pathogens and Splicing Inhibition

Inteins are found in the genomes of archaea, bacteria, single-celled eukaryotes, and viruses, often within proteins required for survival. Here we discuss the mycobacterial inteins within RecA, DnaB, and SufB (Figure 1A), as their splicing is important for the survival of pathogenic mycobacteria. Additionally, we consider Prp8 inteins present in fungal pathogens including *Cryptococcu*s *neoformans*. We also discuss the chemical means by which inhibitors of protein splicing interact with their target intein.

### Mycobacterial Inteins

Mycobacteria are rod-shaped, acid-fast bacteria. Many species are human pathogens, most notably *M. tuberculosis* and *M. leprae*, as well as non-tuberculosis mycobacteria (NTM; i.e. mycobacteria other than *M. tuberculosis* or *M. leprae* that cause disease in humans). Tuberculosis remains a leading cause of death worldwide, causing an estimated 1.4 million deaths in 2019, with multidrug-resistant (MDR) strains becoming increasingly common (Paulus, 2003; World Health Organization, 2020). The World Health Organization has deemed it a priority to address MDR tuberculosis and emerging extensively drug-resistant tuberculosis (Kempker et al., 2015). In 2018, there were ∼209,000 new cases of leprosy, a disease that can cause permanent damage to the skin, nerves, limbs, and eyes if untreated. Emerging drug resistance of *M. leprae* has also been detected (da Silva Rocha et al., 2012). NTM, while less common and generally considered opportunistic pathogens, cause a range of serious diseases and are increasing in incidence (Shamaei and Mirsaeidi, 2021). Mycobacteria are rich in inteins, particularly in three important genes critical for the growth of *M. tuberculosis*: DnaB, SufB, and RecA (Figure 1) (Paulus, 2003). Related mycobacterial species also have inteins present in these same genes, yet the location and sequence can vary. If a small molecule is able to inhibit splicing of these proteins, the mature form of the protein will not be produced and could prevent infection. Therefore, it is of great interest and urgency to investigate inteins within these mycobacterial pathogens as targets for compounds that specifically block splicing. The potential role of inhibiting the splicing of the RecA and DnaB inteins in *M. tuberculosis* has previously been reviewed (Paulus, 2003), and these inteins have subsequently been the subject of investigation both *in vitro* as well as *in vivo* in the mycobacteria.

### Recombinase A Inhibition

**FIGURE 3:**
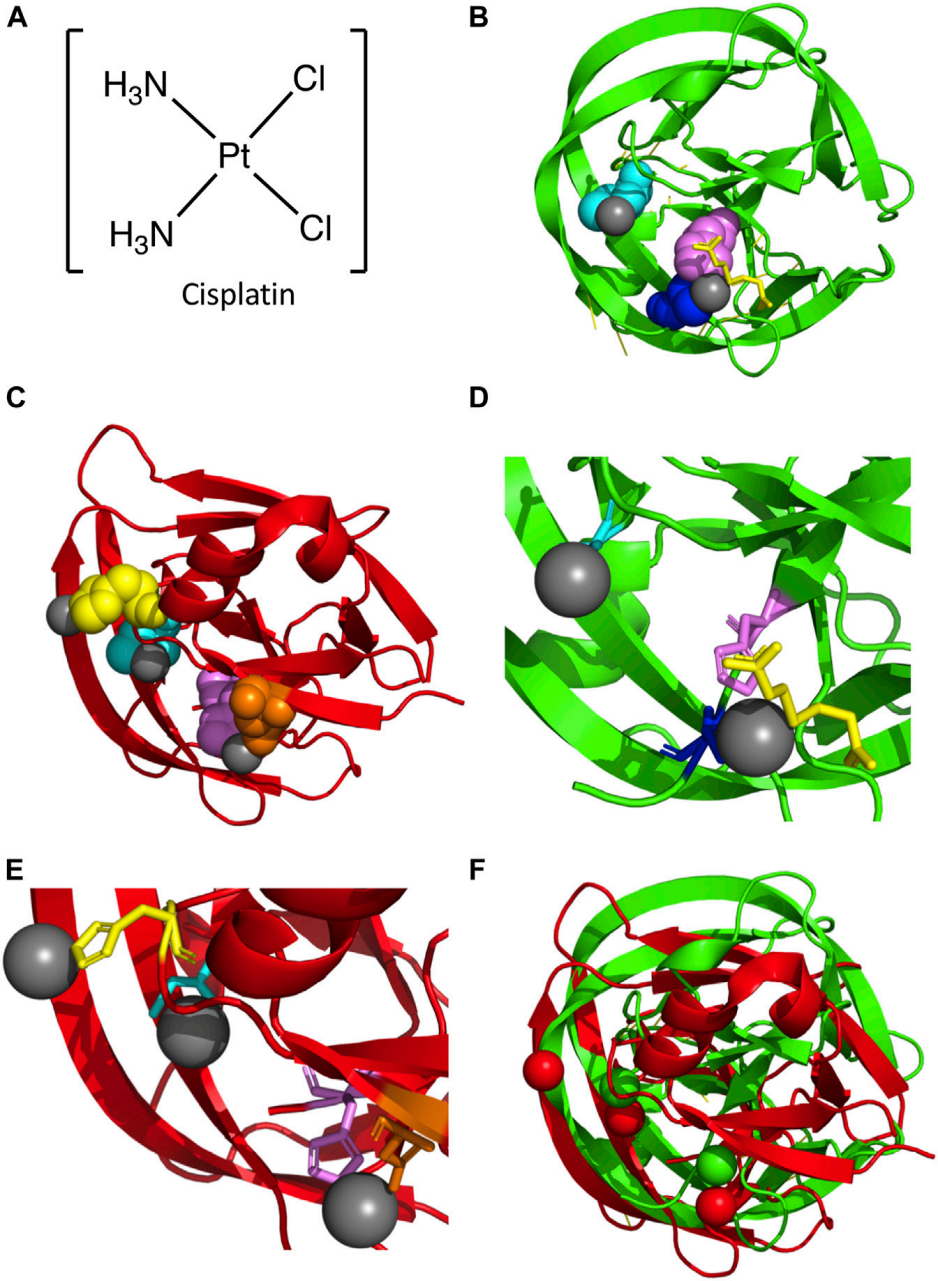
Platinum-bound Intein Structures. **(A)** Structure of cisplatin. **(B)** Structure of the *M. tuberculosis* RecA intein bound to two Pt(II) ions (PDB5I0A; (Chan et al., 2016). Protein backbone in red and Pt(II) in gray. Key residues are colored and represented in space fill, with Cys1 (cyan), His439 (magenta), and Cys+1 (blue) shown. Additionally, TCEP is shown in yellow. **(C)** Structure of the *C. gattii* Prp8 intein in complex with three Pt(II) ions (PDB6MYL; (Li et al., 2019). Protein backbone in red and Pt(II) in gray. Residues important in coordination of Pt(II) are shown as space fill and colored, with Cys1 (cyan), His62 (yellow), Asp94 (orange), and His169 (magenta) shown. **(D)** Zoom in of *M. tuberculosis* RecA intein co-crystal structure bound to two Pt(II). Colored as in **(B)**, with Cys1, His439, and Cys+1 represented as sticks. **(E)** Zoom in of *C. gattii* Prp8 intein structure in complex with three Pt(II). Colored as in panel C, with Cys1, His62, Asp94, and His169 represented as sticks. **(F)** Alignment of the *M. tuberculosis* RecA intein complex with two Pt(II) and the *C. gattii* Prp8 intein bound to three Pt(II). The *M. tuberculosis* RecA intein protein backbone and Pt(II) are colored green, while the *C. gattii* Prp8 intein and Pt(II) are colored red.

One possible mode of mycobacterial inhibition is targeting the intein in DNA Recombinase A (RecA), a protein necessary for the repair and maintenance of DNA in mycobacteria. The *M. tuberculosis* RecA intein is one of the best studied inteins, used in multiple mechanistic studies and several engineering applications (Davis et al., 1991; Davis et al., 1992; Mills et al., 1998; Shingledecker et al., 1998; Frischkorn et al., 2000; Saves et al., 2000; Shingledecker et al., 2000; Hiraga et al., 2005; Van Roey et al., 2007; Zhang et al., 2009; Du et al., 2010; Zhang et al., 2010). It has been found that copper and zinc ions inhibit protein splicing. Copper (II) ions inhibit splicing of a minimized RecA intein by binding to the catalytic Cys1 and block B His residue, and may have a redox effect (Zhang et al., 2010). Zinc ions can also inhibit splicing of the full length and minimized RecA *cis*-splicing inteins as well as a *trans-*splicing variant (Mills and Paulus, 2001; Zhang et al., 2010). Zinc ions have been shown to bind to the C-terminal active site residues of the *Synechocystis* sp. PCC6803 DnaE and *S. cerevisiae* PI-SceI inteins (Poland et al., 2000; Sun et al., 2005). Cisplatin inhibits the splicing of the RecA intein by binding to Cys1, and other platinum-containing compounds are also able to inhibit splicing *in vitro* (Figure 3A) (Zhang et al., 2011). Interestingly, over-expression of the intein was able to reverse the growth inhibition of *Mycobacterium bovis* by cisplatin, suggesting direct binding *in vivo* (Zhang et al., 2011). The crystal structure of a genetically minimized *M. tuberculosis* RecA intein engineered to lack its endonuclease domain (ΔHE domain variant) in complex with cisplatin shows two dissociated platinum ions (Pt(II)) bound to residues required for intein catalysis (Chan et al., 2016) (Figures 3B, D). One Pt(II) binds directly to the catalytic Cys1 residue, with the other coordinated by the highly-conserved penultimate histidine (H439), Cys+1, and the reducing agent (tris(2-carboxyethyl)phosphine (TCEP) (Figures 3B, D). As these Pt(II) are coordinated directly by residues necessary for protein splicing, the mechanism of inhibition is relatively straightforward.

While these compounds can serve as effective inhibitors of *M. tuberculosis* growth through the inhibition of protein splicing, they do so most effectively at micromolar concentrations that may not be suitable for *in vivo* applications, making them more attractive as lead compounds (El-Kareh and Secomb, 2003). Additionally, cisplatin has significant side effects when used as a chemotherapeutic due to its ability to induce DNA damage; such side effects would likely not be tolerated for an antibiotic except perhaps for cancer patients also infected with tuberculosis (Dasari et al., 2015). However, this proof-of-concept inhibition could serve to inspire the development of selective inhibitors that target this intein.

### DnaB Inhibition

DnaB is an essential, intein-containing helicase required for replication. DnaB is also the most common intein-housing gene in bacteria (Novikova et al., 2016). In mycobacterial species housing DnaB inteins, some species contain one intein, while others contain two inteins. *Mycobacterium smegmatis* is a species with two DnaB inteins. The first intein, DnaBi1, is a mini-intein localized to the p-loop of the ATPase domain that splices by an alternative class three mechanism (Kelley et al., 2018). The second intein, DnaBi2, is a full-length, HE domain-containing intein. Based on intein sequence similarity and insertion site, DnaBi1 is similar to an intein from *M. leprae*, while DnaBi2 is similar to an intein from *M. tuberculosis* (Kelley et al., 2018). In *M. smegmatis*, DnaBi1 splicing *in vitro* is reversibly inhibited in oxidizing environments induced by high micromolar concentrations of hydrogen peroxide (Kelley et al., 2018). DnaBi1 splicing inhibition by hydrogen peroxide results in the formation of an intramolecular disulfide bond between the catalytic nucleophile (Cys118) and a structurally-adjacent cysteine (Cys48). Additionally, this intein binds zinc with apparent low micromolar affinity, which also reversibly blocks splicing (Woods et al., 2020). The crystal structure of DnaBi1 bound to zinc shows the ion coordinated by the Cys118, presumably blocking the ability of this catalytic nucleophile to initiate the splicing reaction, as well as with V119 and Y128 (Woods et al., 2020). The *M. leprae* DnaB intein also has been shown to be reversibly inhibited by zinc (Woods et al., 2020). Conversely, DnaBi2 splices quickly without being affected by oxidation.

Importantly, in addition to *in vitro* results, DnaBi1 splicing inhibition by hydrogen peroxide and zinc has been observed within *M. smegmatis* cells using a kanamycin intein splicing reporter (described below) (Kelley et al., 2018; Woods et al., 2020). These results are intriguing, both from the perspective of testing potential antibacterial agents, but also for understanding potential intein-based stress responses as they demonstrate that splicing inhibition by a small molecule can prevent extein function in mycobacteria*.* Interestingly, pathogenic mycobacteria encounter and can withstand hydrogen peroxide and metal stress when encountering mammalian innate immune cells such as neutrophils (Zahrt and Deretic, 2002; Wagner et al., 2005; Neyrolles et al., 2015; Weiss and Schaible, 2015; Gao et al., 2018).

### SufB Inhibition

Another intein of interest interrupts the Fe-S cluster assembly protein B, or SufB. SufB is part of a larger protein complex that functions to repair oxygen-labile iron-sulfur clusters under oxidative stress. As it is highly sensitive to oxidation and nitrosylation, this intein may help regulate SufB function. Splicing of the SufB intein is essential for *M. tuberculosis* and the assembly of the SufBCD complex, an essential system for iron-sulfur cluster assembly in mycobacteria (Huet et al., 2006). The intein Cys1 and Cys+1 residues have low pKa values, which enhances their nucleophilicity and makes them sensitive to oxidation, and thus can prevent splicing in oxidative environments (Topilina et al., 2015a). Environments containing oxidative elements such as hydrogen peroxide and diethanolamine inhibit SufB splicing, resulting in precursor accumulation or irreversible attenuation through N-terminal cleavage. Such oxidative inhibition leads to the formation of a disulfide bond between the catalytic Cys1 and Cys+1 residues, which inhibits normal splicing activity (Topilina et al., 2015a).

### Fungal Inteins

Several human fungal pathogens, such as *Cryptococcus* species*, Ajellomyces capsulatus*, and *Aspergillus* species, contain inteins, most often in the essential spliceosomal Prp8 protein (Green et al., 2019). These fungal species cause pulmonary infections in humans such as cryptococcosis, histoplasmosis, and aspergillosis, respectively, which pose a particular threat to immunosuppressed individuals (Bongomin et al., 2017).

### Pre-mRNA Processing Factor 8 Inhibition

Pre-mRNA processing factor 8 (Prp8) is a highly conserved nuclear protein ranging in size from 230 to 270 kDa. Prp8 has been identified as an essential mRNA splicing factor involved early in the mRNA splicing process (Grainger and Beggs, 2005). Prp8 is located in the catalytic core of the spliceosome complex, where pre-mRNA splicing occurs. In the spliceosome, Prp8 is a component of the U5 small nuclear ribonucleoprotein (sNRP) and U5•U4/U6 tri-sNRP and is believed to act as a cofactor in RNA catalysis.

Over 100 Prp8 inteins have been identified. There are both HE-containing inteins and mini-inteins, varying from 150 to over 800 residues (Butler et al., 2006). Many of the inteins occupy the same insertion site, with a total of seven insertion sites identified (Green et al., 2019). Phylogenetic analysis of these inteins suggests that the first inteins were introduced into Prp8 over a billion years ago, with the most recent being introduced approximately 400 million years ago. Additionally, the inteins are phylogenetically clustered both by insertion site and by organism, suggesting that multiple, independent invasion events occurred throughout the evolution of this protein (Green et al., 2019).

The Prp8 intein is an exciting potential drug target given the essential role for this protein in RNA splicing (Liu and Yang, 2004). Divalent metals such as zinc and copper ions can inhibit the splicing of the *C. neoformans* intein. Though they do so in different ways, they both function by interfering with the catalytic N-terminal Cys1 of the intein. Copper ions reversibly oxidize the thiol of Cys1. Zinc ions have two intein binding sites, one at the C-terminal asparagine residue and the other at Cys1; binding at the Asn site may be tighter (Green et al., 2019). Similarly, cisplatin can bond with Cys1 and interfere with its activity (Li et al., 2019). The Prp8 intein of *C. neoformans* can be targeted by cisplatin, as with the *M. tuberculosis* RecA intein, which provides protection to mice from infection (Li et al., 2019). The cisplatin binds similarly at Cys1 in the Prp8 intein as it does in the RecA intein; however, a second cisplatin that binds at a downstream conserved active-site His residue may be more tightly coordinated by a second ligand (Li et al., 2019). The co-crystal structure of the *C. gattii* Prp8 intein with cisplatin (Li et al., 2019) has three dissociated Pt(II) molecules, as opposed to two Pt(II) for *M. tuberculosis* RecA (Figure 3C). As with *M. tuberculosis* RecA, one Pt(II) is bound to the catalytic Cys1 residue. Additionally, another Pt(II) is coordinated by the conserved penultimate histidine (H169) similar to *M. tuberculosis* RecA (Figures 3C, F). Unlike *M. tuberculosis* RecA, the *C. gattii* Prp8 intein binds a third Pt(II) at a non-conserved histidine (His62) (Figures 3C, F). Overall comparison with *M. tuberculosis* RecA shows considerable similarities in the overall alignment of the two structures (Figure 3D). In particular, the coordination of Pt(II) at both N- and C-terminal residues required for intein activity (Figure 3D). Furthermore, as with *M. smegmatis* DnaBi1(Woods et al., 2020), the inhibitor is bound directly by residues strictly required for protein splicing, explaining the mechanism of inhibition.

Recently, a landmark study on the use of inhibitors that target Prp8 protein splicing as a strategy to selectively kill pathogenic fungi was published (Li et al., 2021). In this work, small molecules identified from a library that inhibit the splicing of the Prp8 intein *in vitro*, in *E. coli* and in *C. neoformans*, reduced the growth of intein-containing *C. neoformans* and *C. gattii*, but not of the intein-less *Candida albicans* (Li et al., 2021). The inhibition is likely via a covalent modification to Cys1 of the Prp8 intein. This is a particularly compelling development in the exploration of inteins as novel antimicrobials, strongly supporting the viability of this strategy.

## Screening for Intein Activity

Selective small molecule inhibitors of protein splicing could be effective tools against several human pathogens. Ideally, a system to screen for selective inhibitors would be high-throughput and could assay against inhibition of off-site targets or general cytotoxicity. Although many existing drugs are covalent mechanism-based inhibitors, it would be important to select for drugs specific to the intein rather than simply to a reactive Cys or Ser to prevent off-site toxicity. Selectable systems have been employed in directed evolution experiments to tune intein function, and split exteins have been used as reporters for splicing activity (Shah and Stevens, 2020). Several of these systems could be repurposed for drug screening. Below, we describe systems based on kanamycin resistance, GFP fluorescence and luciferase activity, CcdB/CcdA activity, thymidylate aynthase activity, and β-galactosidase colorimetry.

### Aminoglycoside Phosphotransferase

**FIGURE 4:**
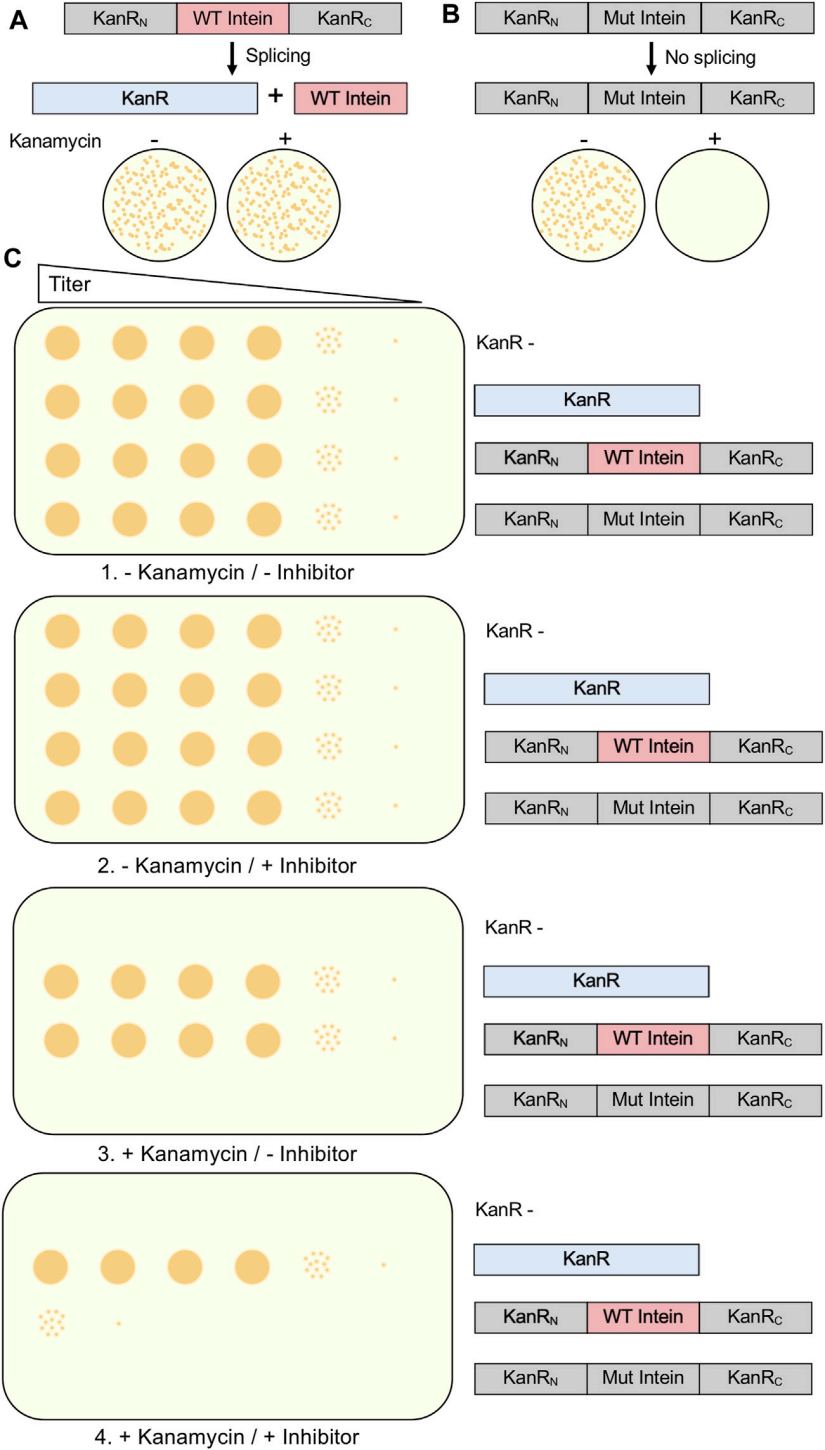
Kanamycin Intein Splicing Reporter. For a splicing-dependent reporter, KanR interrupted with a catalytically active intein is resistant to kanamycin **(A)**, while KanR interrupted with a catalytically inactive intein that cannot undergo splicing is not resistant to kanamycin **(B)**. In **(C)**, we show the experimental design to test the specific inhibition of protein splicing using a kanamycin-intein splicing reporter. In addition to cells containing the splicing-dependent KanR-intein fusion plasmid, control plasmids include KanR without an intein, a splicing inactive KanR-intein fusion, and kanamycin cells lacking a KanR-containing plasmid. In this design, serial dilutions of cells should be grown under four conditions: 1) without kanamycin and without splicing inhibitor; 2) without kanamycin and with splicing inhibitor; 3) with kanamycin and without splicing inhibitor; and 4) with kanamycin and with splicing inhibitor. Growth of the kanamycin sensitive and splicing inactive KanR-intein fusion strains should only occur in the absence kanamycin. Presence of the splicing inhibitor in the absence of kanamycin should not decrease growth of any strain. If inhibition of protein splicing occurs, the splicing-dependent KanR-intein fusion will display reduced survival compared to KanR without an intein. Interrupted, inactive exteins are colored gray, inteins are colored pink if active and gray if inactivated by mutation, and active KanR is colored blue.

One method used to evaluate intein splicing and therefore serve as a screen for small molecule inhibitors is the use of the kanamycin resistance protein interrupted by an intein. Kanamycin A is an aminoglycoside antibiotic that inhibits translation and is widely used in molecular biology. Bacterial cells able to grow on media containing the antibiotic require a mature aminoglycoside phosphotransferase (KanR). Experimental cells are transformed to contain the KanR gene interrupted by an intein. Typically, the positive control is the uninterrupted KanR gene without an intein, as no splicing is required to produce resistance to kanamycin. When designing a kanamycin intein splicing reporter for the purpose of evaluating splicing inhibitors, it is crucial that protein splicing be required to provide resistance (Figure 4A). Somewhat surprisingly, when splicing active and inactive versions of the *M. smegmatis* DnaBi1 intein were inserted at 16 positions throughout the KanR protein, five displayed splicing-dependent resistance, eight displayed splicing-independent resistance, and three provided no resistance (Woods et al., 2020). Therefore, to ensure that kanamycin resistance cannot be provided by a KanR protein that is still interrupted by an intein, which would result in false positives when selecting for intein activity or potentially false negatives when searching for splicing inhibitors, a splicing inactive intein must also be tested at the same position within the KanR gene (Kelley et al., 2018; Woods et al., 2020; Woods et al., 2021). As expected, proximity to the active site correlated with the requirement of splicing for resistance, with all *M. smegmatis* DnaBi1-KanR splicing-dependent resistance fusions were within 14 residues and 16 Å of the active site (Woods et al., 2020). In addition to ensuring that the inhibition of splicing would lead to kanamycin sensitivity by blocking splicing, the potential splicing inhibitor must be tested for cytotoxicity independent of splicing and lack of kanamycin resistance (Kelley et al., 2018; Woods et al., 2020) (Figure 4B). Ideally, the intein-KanR fusion should display reduced survival relative to an uninterrupted KanR in the presence of both a potential inhibitor and kanamycin (Figure 4B).

A significant advantage of the kanamycin intein splicing reporter is that it has been shown that it can be used in the native intein host, in this case *M. smegmatis*, so long as the host is sensitive to kanamycin. This system has been used in *M. smegmatis* to detect inhibition of the DnaBi1 intein by hydrogen peroxide and zinc (Kelley et al., 2018; Woods et al., 2020; Woods et al., 2021), both stressors mycobacteria can encounter during challenge from the innate immune system (Zahrt and Deretic, 2002; Wagner et al., 2005; Neyrolles et al., 2015; Weiss and Schaible, 2015; Gao et al., 2018). Although *M. smegmatis* is generally non-pathogenic, and results may not translate exactly to mycobacteria that harbor conserved inteins like *M. tuberculosis* and *M. leprae*, it is useful as proxy for these pathogens. Importantly, this general approach could be adapted to any intein-containing pathogen, as long as the pathogen is sensitive to kanamycin and appropriate expression vectors are available. On the other hand, one disadvantage to this screening system is the possibility of missing a lead compound that could serve as an inhibitor because it cannot penetrate the cells. Additionally, adaptation of the system to a high-throughput liquid culture format, rather than agar plates, could significantly increase the speed at which potential inhibitors can be tested.

It should be noted that the KanR-intein fusion design has been used in applications other than to search for inhibitors. One of the first uses of a kanamycin screening system was to identify open reading frames of the pathogen *Haemophilus influenzae* (Daugelat and Jacobs, 1999). In another application, a kanamycin selection system was used to make splicing conditional on addition of a small molecule, 4-hydroxytamoxifen (Buskirk et al., 2004). Finally, a KanR system has been used to improve splicing of the naturally split *Nostoc punctiforme* DnaE intein through directed evolution (Lockless and Muir, 2009).

### Green Fluorescence Protein and Luciferase

**FIGURE 5:**
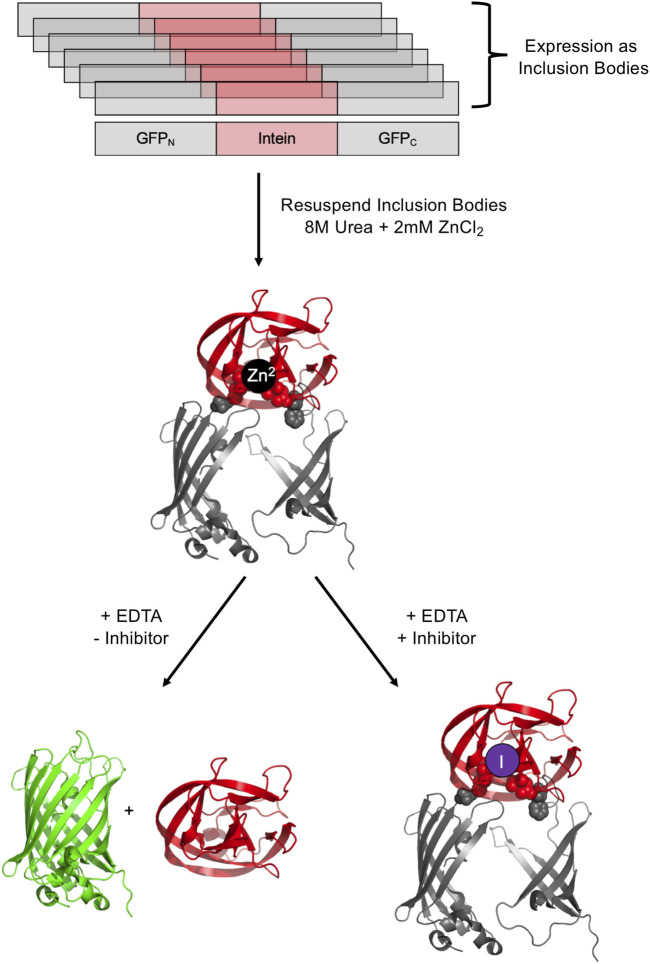
GFP-intein fusion fluorescence reporter. The GFP-intein fusion is expressed as inclusion bodies (as indicated by overlapping diagrams), denatured by urea, and renatured in the presence of Zn^2+^ to prevent splicing. To allow splicing to proceed, EDTA is added to chelate Zn^2+^, yielding fluorescent GFP and the excised intein. In the presence of a splicing inhibitor of interest, splicing is blocked, leading to no increase in fluorescence. Exteins are colored gray and the intein is colored pink/red. Post-splicing GFP is colored green. Zn^2+^ is colored black and inhibitor purple. PDBs 2B3P (Superfolder GFP) and 2IN9 (*M. tuberculosis* RecA mini intein) were used to create this image (Pedelacq et al., 2006; Van Roey et al., 2007).

It is possible to screen for intein activity by making the fluorescence of green fluorescent protein (GFP) conditional on protein splicing either in *cis* and in *trans*. GFP fluorescence is useful for small molecule inhibitor discovery as it is amenable to high throughput screening and does not require potential lead compounds to penetrate a cell. The full-length *M. tuberculosis* RecA intein was used to interrupt GFP after residue 129, based on the observation that peptide insertions adjacent to this residue result in GFP expression into inclusion bodies in *E. coli* (Figure 5) (Gangopadhyay et al., 2003). An N-terminal His tag allows for purification of intein-interrupted GFP via immobilized metal affinity chromatography, followed by denaturation in 8M urea and renaturation into a folding buffer. The addition of zinc ions during refolding prevents intein activity as measured by GFP fluorescence, and this inhibition can be readily reversed by the addition of EDTA (Gangopadhyay et al., 2003). *In vitro* screening may be facilitated by mixing potential inhibitors during the refolding process. GFP fluorescence was used in a directed evolution experiment to measure splicing efficiency of evolved versions of the *M. tuberculosis* RecA intein (Yuen et al., 2006). The *Synechocystis* sp. PCC6803 DnaE split intein was used to reconstitute GFP in an assay to detect protein-protein interactions; the intein was reported to facilitate some GFP fluorescence without other interacting partners driving the association and hence this could serve as a means to select inhibitors for DnaE split intein reassociation (Ozawa et al., 2001).

GFP fluorescence as a screening system was used to explore the effect of platinum-containing compounds on the splicing of a minimized *M. tuberculosis* RecA intein (Chan et al., 2016). These compounds, including cisplatin, Pttfbz, Zeise’s salt, and phenanthriplatin, were added to a stabilized precursor GFP-RecA fusion protein in concentrations up to 80 µM. Once splicing was initiated with EDTA, the resulting fluorescence was observed, and it was found that increased concentrations of the Pt-containing compounds led to decreased fluorescence, suggesting that the compounds could successfully inhibit RecA protein splicing (Chan et al., 2016). This supported previous work suggesting that cisplatin can be toxic to *M. tuberculosis*, with intein over-expression decreasing the toxicity, indicating direct binding of the intein to cisplatin *in vivo* (Zhang et al., 2011). GFP screening also was used to investigate the influence of mutations of non-catalytic sites on RecA intein activity (Hiraga et al., 2009).

Using a similar design by which interruption with an intein blocks reporter function, the luciferase from *Renilla reniformis* was split between residues 229 and 230 with the Prp8 intein from *C. gattii* (Li et al., 2019). The fusion protein was isolated at pH 9, where splicing was inefficient, and then incubated with or without inhibitor at pH 8, with a decrease in fluorescence measuring splicing inhibition, in this case with cisplatin (Li et al., 2019). Both this split luciferase system, as well as a split GFP system, was used to measure the splicing of the *C. neoformans* Prp8 intein (Li et al., 2021). GFP fluorescence was dependent on TCEP, showing that splicing and thus fluorescence was dependent on the intein active site Cys being present as a free thiol. The system also allowed for discovery of small molecule inhibitors of the intein, as discussed above (Li et al., 2021).

### CcdA/B Toxin-Antitoxin

Another in-cell screening system utilizes the *E. coli* toxin CcdB, which targets the topoisomerase GyrA (Bernard and Couturier, 1992). The full length *M. tuberculosis* RecA intein was used to interrupt CcdB on both a low and high copy number plasmid, such that cell growth is coupled to splicing inhibition. The intein-ccdB fusion was used to determine splicing efficiency as modulated by mutations to flanking extein residues (Lew and Paulus, 2002). Using plasmids with variable copy number and/or expression levels using a tunable promoter could vary the stringency used in different rounds of small molecule screening. A similar screening system utilizes the *E. coli* antitoxin CcdA, which acts as the CcdB antitoxin, forming an inactivated complex. In this method, an intein-less CcdB is expressed by an *E. coli* strain, and the intein is instead inserted into the CcdA protein in a region that directly interacts with CcdB, allowing CcdA to only inactivate CcdB if protein splicing occurs. This system worked with the *cis*-splicing *Nostoc punctiforme* DnaB intein and the naturally split *trans*-splicing cyanobacterial gp41-1 intein (Beyer and Iwai, 2019). The CcdA system can serve as an *in vivo* counter-selection method for the CcdB model, as effective intein inhibitors would cause cell death rather than permit cell growth.

### Thymidylate Synthase

**FIGURE 6:**
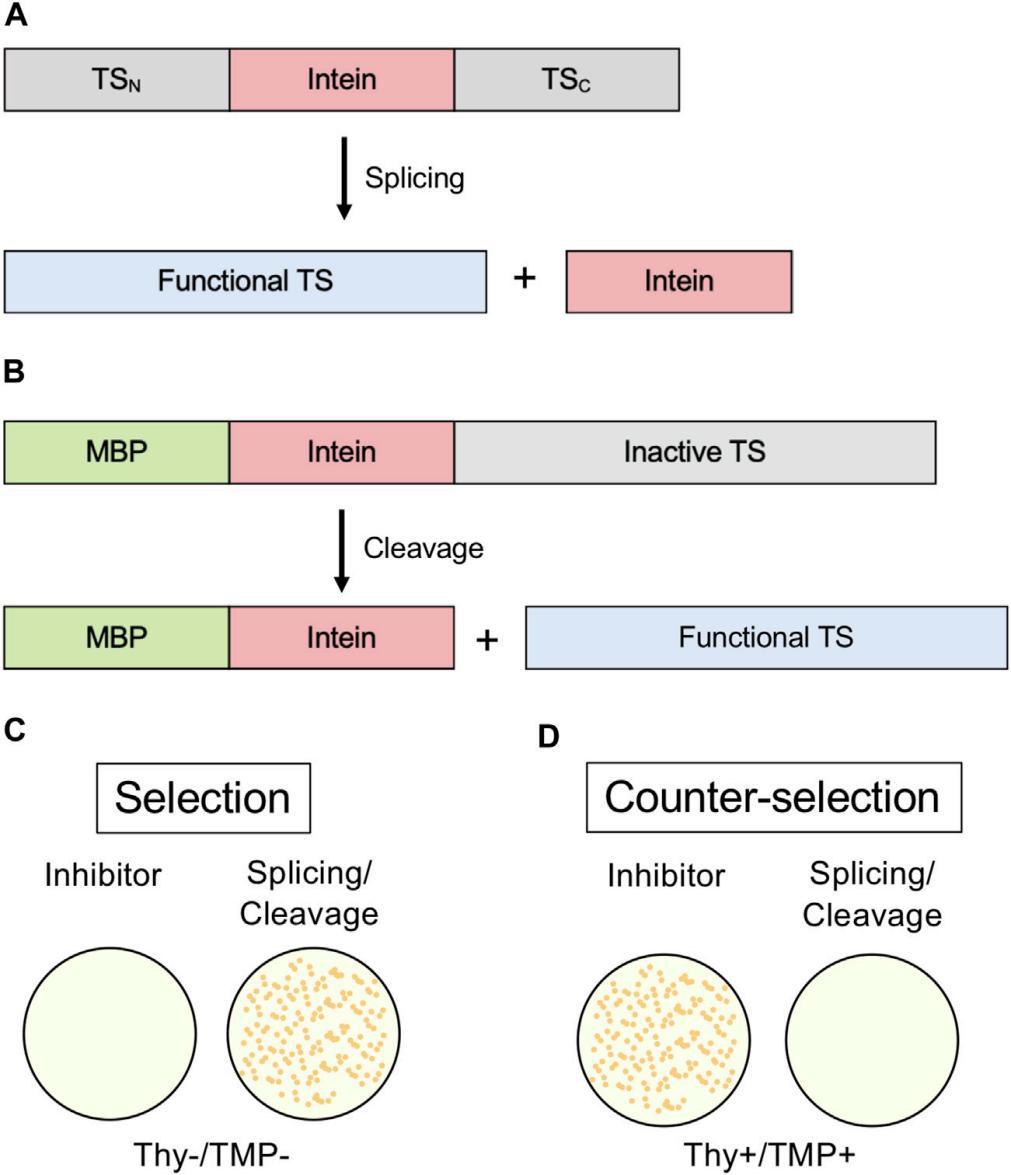
Thymidylate Synthase (TS) selection/counter-selection system. **(A)** Splicing reporter where TS is interrupted by an intein and protein splicing must occur to yield a functional enzyme. **(B)** Cleavage reporter where a variant intein that can only undergo C-terminal cleavage interrupts an N-extein of maltose-binding protein (MBP) and a C-extein of TS. Because N-terminal fusions disrupt TS activity, C-terminal cleavage of the intein must occur to yield a functional enzyme. **(C)** TS activity is required for growth to occur in thymidine-lacking media (Thy-). **(D)** In the counter-selection model, functional TS inhibits growth in the presence of trimethoprim (TMP). Exteins are colored gray, intein pink, MBP green, and active TS blue.

An intein-thymidylate synthase (TS) fusion represents another *in vivo* system that may be used to monitor protein splicing. *E. coli* in the absence of thymidine in media cannot grow without functional TS; therefore, TS can serve as an indicator of splicing when it is interrupted by an intein (Wood et al., 1999). *E. coli* that lack their native TS can be transformed with a plasmid coding for TS with an intein inserted. If the transformed bacteria are grown in thymidine-lacking media, growth will not occur without protein splicing, as the unspliced intein-TS fusion protein is nonfunctional (Figure 6A). This system conveniently has a natural negative control, as growth in thymidine-rich media would not be splicing-dependent, and an inhibitor should show little effect on cell growth if its activity is intein-specific. Another useful aspect of this system is that N-terminal fusions inactivate TS (Figure 6B). Therefore, intein-mediated splicing and C-terminal cleavage can also be monitored by this system (Figure 6C) (Wood et al., 1999). This system was used to isolate an activating splicing mutant of a minimized version of the *M. tuberculosis* RecA intein and to select a mutant that facilitates pH-dependent C-terminal cleavage (Wood et al., 1999).

The TS system also has been used to design an intein-based system by which the miniaturized *M. tuberculosis* RecA intein was interrupted by a thyroid hormone receptor, making splicing and TS activity sensitive to the addition of thyroid hormone (Skretas and Wood, 2005). A TS-splicing system, in a modified format, also has been used to screen libraries of *de novo* protein sequences for frameshifts or stop codons. Insertion of gene sequences upstream of the intein-TS fusion that are out of frame prevent expression of the intein-TS fusion and hence growth on thymidine-free media (Bradley et al., 2005).

Screening for intein activity by conditional TS activity also has the advantage of a counter-selection system, using trimethoprim (TMP) (Figure 6D) (Wong et al., 2005). TMP inhibits dihydrofolate reductase and therefore prevents the production of tetrahydrofolate (THF). If TS is active, THF is depleted. In thymidine-rich media, the cells don’t require active TS for thymidine and can grow, but active TS is toxic in the presence of TMP. This model of counter selection allows for screening of inhibitors of splicing or C-terminal cleavage.

### β-galactosidase

**FIGURE 7:**
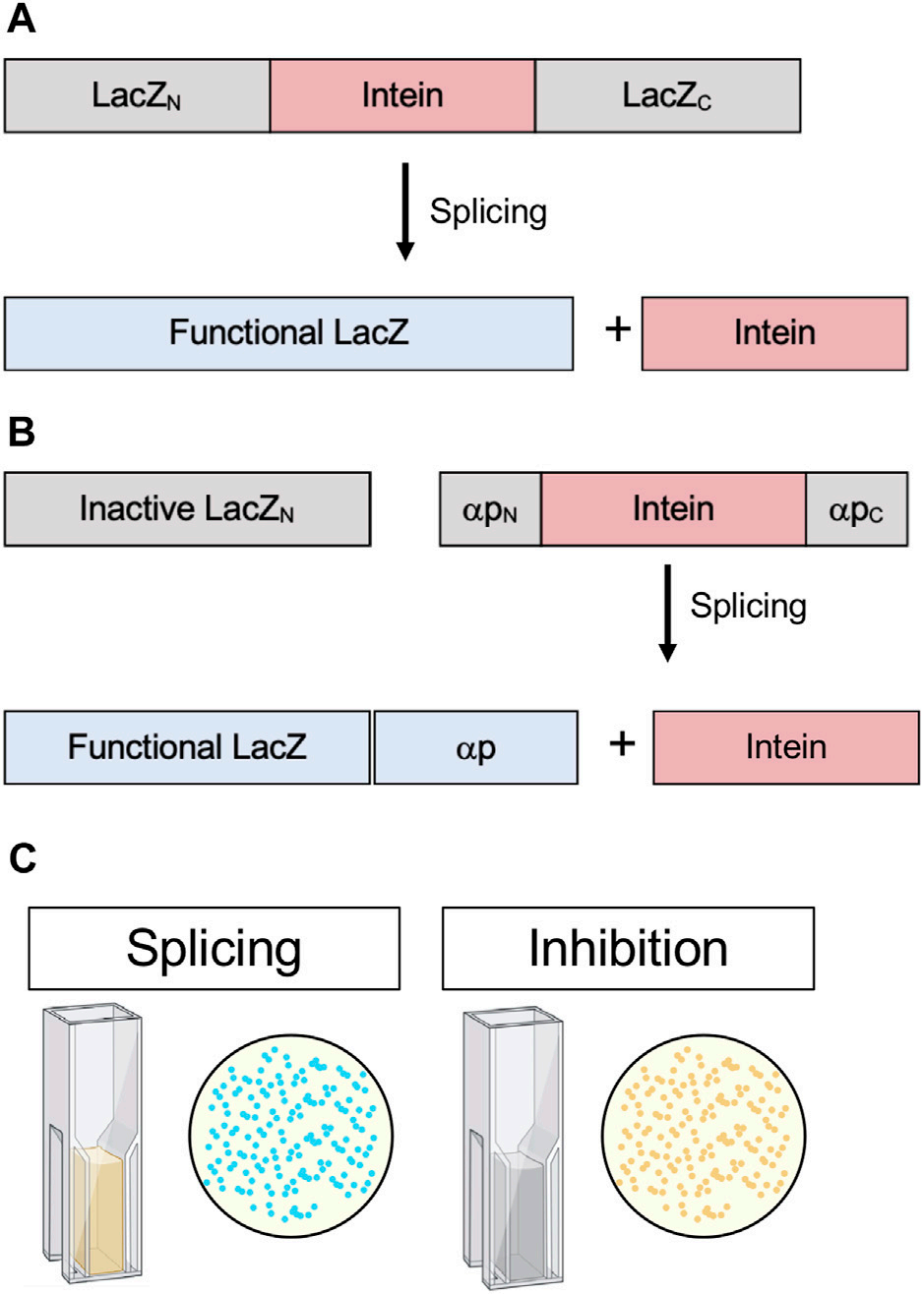
. β-galactosidase (*lacZ*) splicing and cleavage reporters. **(A)** Splicing reporter where β-galactosidase/LacZ is interrupted by an intein and protein splicing must occur to yield a functional enzyme. **(B)** Splicing reporter where β-galactosidase/LacZ is split and the α-peptide, which complements enzyme activity, is interrupted by an intein and protein splicing must occur for the α-peptide to restore β-galactosidase/LacZ activity. **(C)** β-galactosidase/LacZ activity can be detected in colonies, indicated by blue color, or quantitatively measured in cell lysates by Miller Units (yellow color). Exteins are colored gray, intein pink, and active β-galactosidase/LacZ blue.

β-galactosidase is a widely employed *E. coli* reporter. Functional β-galactosidase cleaves X-gal into 5-bromo-4-chloro-3-hydroxyindole, which spontaneously dimerizes and oxidizes to form the bright blue pigment 5,5’-dibromo-4,4’-dichloro-indigo. Active β-galactosidase in *E. coli* results in blue colonies, whereas inactive β-galactosidase results in white colonies. In a commonly used technique β-galactosidase is split, and insertions into the α-peptide, which complements enzyme activity, can be used for blue-white screening (Green and Sambrook, 2019). If colonies are white, an insertion has been made in the α-peptide and β-galactosidase is nonfunctional. Additionally, β-galactosidase activity can be assayed quantitatively from cell lysates by measuring Miller Units, another colorimetric assay that measures the conversion of *o*-nitrophenyl-β-d-galactoside to galactose and *o*-nitrophenol, the latter which can be measured spectroscopically (Smale, 2010). Modified blue-white screening has been used by interrupting *E. coli* β-galactosidase with an intein. For example, this was used to analyze the splicing activity of an estrogen-sensitive version of the *S. cerevisiae* vacuolar ATPase (VMA) intein in the presence of estrogen or synthetic analogs (Figure 7A). Rather than inhibition, this system tested for activation of the intein (Liang et al., 2011).

Another version of blue-white screening was used to identify active intein mutants and characterize the conditions favorable to splicing. Modified and artificially split versions of the *Synechocystis sp.* PCC6803 DnaB intein were inserted into two different sites in the β-galactosidase α-peptide whereby complementation of enzyme activity was dependent on splicing (Figure 7B). Subsequently, this system were used to test the influence of active site mutations (Neugebauer et al., 2017). This time-tested assay could serve as a powerful way to screen for *in vivo* intein inhibition in a scalable model that does not rely on cytotoxicity as an output, as well as provide the option for quantitative measurements via Miller Units (Figure 7C).

## Hedgehog Autoprocessing and Off-Target Considerations

The Hedgehog (Hh) signaling pathway is required for cell differentiation and proliferation during animal development. In adults, Hh signaling is mostly turned off but is involved in adult tissue regeneration and stem cell maintenance. Abnormal Hh activation is implicated in many types of cancer (Sigafoos et al., 2021). Because the structure and mechanism of inteins and the Hh family of proteins share so many features (Hall et al., 1997), it is important to consider the potential effect of intein inhibitors on Hh signaling, including Hh autoprocessing.

Hh ligands are produced from a unique self-catalyzed process, called Hh autoprocessing. The Hh ligand is not a direct product of translation; rather, it comes from an Hh precursor protein, which is subject to two post-translational modifications: cholesterylation at the C-terminus of the Hh ligand during Hh autoprocessing, and subsequently palmitoylation at the N-terminus (Callahan and Wang, 2015). Due to increased hydrophobicity, these post-translational modifications help establish the Hh gradients necessary for Hh signaling in development, and abnormalities in the post-translational modifications can lead to severe congenital diseases, including holoprosencephaly (HPE) (Roessler et al., 1997; Incardona and Roelink, 2000).

**FIGURE 8:**
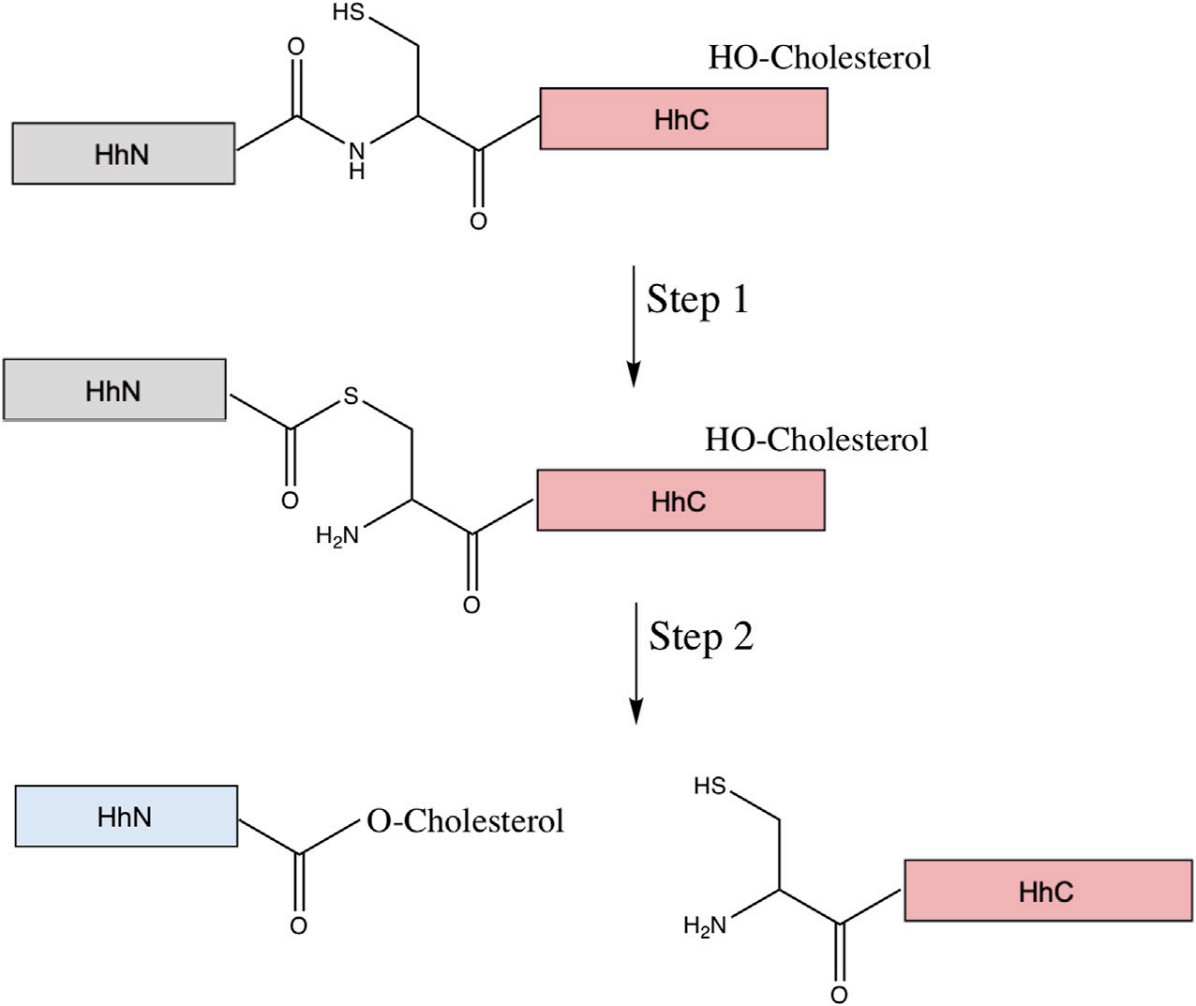
Mechanism of hedgehog autoprocessing. The hedgehog precursor protein is comprised of the signaling domain (HhN, gray) and the autoprocessing domain (HhC in pink, comprised of a HINT domain (Hedgehog-INTein) and a C-terminal sterol recognition region (SRR). The first step of the autoprocessing is similar to that of protein splicing: conversion of the peptide bond linking the HhN to HhC from an amide to thioester via the N-terminal Cys of the HhC domain. Then, in place of the trans-thioesterification via the C-extein that occurs in protein splicing, a cholesterol molecule, bound to the SRR, facilitates an addition/elimination reaction at the newly formed thioester, resulting in cholesterylation of HhN concomitant to bond cleavage between HhN and HhC. The HhN (now blue) is further modified by N-terminal palmitoylation prior to its function in signaling.

The Hh precursor consists of two domains: the N-terminal domain, HhN, and the C-terminal domain, HhC. HhN is the signaling domain, or Hh ligand. The HhC is the autoprocessing domain, consisting of the HINT domain and the sterol-recognition region (SRR). The HINT region shares many structural and functional similarities to the protein splicing of inteins (Xie et al., 2016). Hh autoprocessing consists of two steps (Figure 8). The first step is an N-S acyl shift, an amide to thioester rearrangement of the peptide bond linking HhN and HhC, identical to the first step of protein splicing. The second step is a transesterification using cholesterol as the nucleophile, resulting in cleavage of the thioester bond linking HhN and HhC and the cholesterylation of the C-terminus of HhN (Callahan and Wang, 2015; Xie et al., 2016). The precise means by which the SRR binds and activates cholesterol is unclear, but it is possible that the SRR may tether the entire Hh to a cholesterol-rich membrane, facilitating the recruitment of the cholesterol directly to the HhN active site, that it extracts cholesterol from the membrane and moves it to the Hh active site, or that it provides a hydrophobic conduit (Mafi et al., 2021). Next, the cholesterylated HhN is further lipidized by the hedgehog acyl transferase, which covalently links palmitic acid to the HhN N-terminus (Pepinsky et al., 1998). Once both lipids are bonded to HhN, the newly formed Hh ligand can be properly transported and used for downstream signaling. The cleaved HhC, on the other hand, is likely degraded by the proteosome (Briscoe and Thérond, 2013).

A mature N-terminally palmitoylated and C-terminally cholesterylated Hh ligand is then secreted into the extracellular space with the help of Dispatched-1 and Scube2. Once in the extracellular space, Scube2 proteins act as chaperones and bind to the Hh ligand during its transfer from producing to receiving cells. The ligand binds to Patched-1 on the receiving cell surface, which then releases its inhibition of smoothened (SMO), activating glioma-associated oncogene homolog (GLI) transcription factors to trigger subsequent signaling events such as development and patterning (Qi and Li, 2020).

As both inteins and Hh family proteins have HINT domains, they share conserved residues. The first is the catalytic Cys1 residue, the nucleophile in the amide-thioester rearrangement. Two other conserved residues, a Thr and His in the TXXH motif of “block B” of the HINT domain, catalyze the thioester formation during autoprocessing. Two additional conserved HhC residues, an Asp and a downstream Cys (D46 in C143 Drosophila HINT), do not have direct homologous residues in intein sequences. The downstream Cys is capable of forming an internal disulfide bond with Cys1, suggesting a role in proper folding (Chen et al., 2011). The conserved Asp likely coordinates the binding of cholesterol to the SRR with the first step of autoprocessing, and activates the hydroxyl group of cholesterol to attack the thioester (Hall et al., 1997; Xie et al., 2016). Surprisingly, substitution of this Asp with His, a nominal charge reversal substitution (D46H in Drosophila HINT), can allow for a wider array of molecules, including coprostanol and epicoprostanol, to serve as the sterol substrate (Zhao et al., 2019). This is likely due to the more favorable, less spatially restrictive electrostatic stabilization of transition state provided by the H46 variant.

Hh autoprocessing can be inhibited by zinc and, similarly to protein splicing, zinc ions can bind to residues vital for autoprocessing to inhibit the function. For *Drosophila* Hh, micromolar zinc concentrations can inhibit autoprocessing, potentially by coordinating Cys1 and the essential His and Asp residues. Prostate, lung, and ovarian cancers are partially characterized by high levels of Hh ligand and low zinc levels, and zinc inhibition of autoprocessing might suggest a link between these observations (Xie et al., 2016). Given that inteins and Hh proteins share zinc-mediated inhibition, some intein-inhibitors may also inhibit Hh autoprocessing, and selective intein inhibitors may be useful. Hh autoprocessing also can be inhibited by selective alkylation of the downstream Cys residue. As measured by a FRET-based cholesterolysis assay, novel compound ST044643 covalently modifies the Cys to inhibit cholersterolysis (Owen et al., 2015). Compounds that activate hydrolysis of the scissile peptide bond between the signaling domain and the autoprocessing domain have recently been described (Smith et al., 2020). These so-called HhC activator compounds redirect the cholesterylation reaction toward hydrolysis, even in the presence of sterol, in a non-covalent mechanism termed paracatalysis. This is exciting, as it suggests that similar paracatalytic compounds may be discovered for inteins, diverting splicing to side reactions that prevent ligation of the flanking exteins. However, this also cautions us that such compounds might be cross-reactive with Hh autoprocessing domains.

Because of the shared HINT domain between intein and Hh autoprocessing, it is important to test if an intein inhibitor is also an inhibitor for Hh autoprocessing. Then, intein inhibitors can be classified into two categories: *selective intein inhibitors* that do not affect Hh autoprocessing and *nonselective inhibitors*, such as Zn ion, that inhibit both intein splicing and Hh autoprocessing. Both types of inhibitors may be useful in clinical settings. For example, if a patient has both Hh ligand-dependent cancer and tuberculosis infection, a nonselective intein inhibitor may be effective against both the cancer and infection, respectively. By inhibiting Hh autoprocessing, the drug will reduce Hh ligand, curbing cancer growth. By inhibiting intein splicing, key DNA enzymes in *M. tuberculosis*, such as the intein-interrupted RecA, cannot be generated, curbing the tuberculosis infection. Indeed, many cancer patients are immunocompromised and more susceptible to TB infection, thus such a clinical scenario can indeed arise. Selective inhibitors will be useful for cases where active Hh signaling needs to be preserved during the application of intein inhibitor, for example in treating TB during pregnancy. To distinguish between selective intein inhibitors and those that also target Hh autoprocessing, screens could be designed in tandem. For example, by combining one of the intein activity reporters described above with a FRET-based cholesterolysis assay for Hh activity (Owen et al., 2015), selective intein inhibitors could be identified.

## Conclusion

Numerous pathogenic bacteria and fungi, including *M. tuberculosis* and *C. neoformans*, harbor inteins within essential genes. Here, we argue that blocking protein splicing, and thus compromising the function of these proteins, provides a potential avenue to kill these devastating pathogens. Decades of intein research has demonstrated that protein splicing can be modulated by numerous environmental factors (Belfort, 2017; Lennon and Belfort, 2017). Given the overall abundance of inteins in the microbial world, presence of inteins in essential genes of bacterial and fungal pathogens, and absence of inteins in metazoans, inteins represent attractive drug targets. While the FDA-approved chemotherapeutic cisplatin has been shown to block protein splicing of inteins from both *M. tuberculosis* and *C. neoformans* (Zhang et al., 2011; Chan et al., 2016; Li et al., 2019), it may not be an appropriate treatment option given off-target DNA damage. However, cisplatin and other splicing inhibitors (Li et al., 2021) demonstrate the proof of concept that splicing inhibitors can be used to kill pathogens and may serve as lead compounds to future drug development. As tuberculosis is typically the leading cause of death worldwide from infectious disease, and multidrug-resistance is becoming increasingly common, there is an urgent need for novel drug targets. The next stages of the development of intein inhibitors as drugs include the validation of inteins as important drug targets by clear demonstration that protein splicing is essential to pathogen survival and/or growth, and attention to developing more complex inhibitors that are specific to inteins rather than closely related hedgehog proteins or somewhat related enzymes such as proteases with reactive active site nucleophiles.
